# Ca_V_3.1 channels facilitate calcium wave generation and myogenic tone development in mouse mesenteric arteries

**DOI:** 10.1038/s41598-023-47715-3

**Published:** 2023-11-21

**Authors:** Mohammed A. El-Lakany, Nadia Haghbin, Naman Arora, Ahmed M. Hashad, Galina Yu. Mironova, Maria Sancho, Robert Gros, Donald G. Welsh

**Affiliations:** 1 Department of Physiology & Pharmacology, Schulich School of Medicine, Robarts Research Institute, University of Western Ontario, 1151 Richmond Road N, London, ON N6A 5B7 Canada; 2 Department of Pharmacology and Toxicology, Faculty of Pharmacy, Alexandria University, Alexandria, Egypt; 3 Department of Physiology, Faculty of Medicine, Complutense University of Madrid, Madrid, Spain

**Keywords:** Cardiovascular biology, Physiology, Blood flow

## Abstract

The arterial myogenic response to intraluminal pressure elicits constriction to maintain tissue perfusion. Smooth muscle [Ca^2+^] is a key determinant of constriction, tied to L-type (Ca_V_1.2) Ca^2+^ channels. While important, other Ca^2+^ channels, particularly T-type could contribute to pressure regulation within defined voltage ranges. This study examined the role of one T-type Ca^2+^ channel (Ca_V_3.1) using C57BL/6 wild type and Ca_V_3.1^−/−^ mice. Patch-clamp electrophysiology, pressure myography, blood pressure and Ca^2+^ imaging defined the Ca_V_3.1^−/−^ phenotype relative to C57BL/6. Ca_V_3.1^−/−^ mice had absent Ca_V_3.1 expression and whole-cell current, coinciding with lower blood pressure and reduced mesenteric artery myogenic tone, particularly at lower pressures (20–60 mmHg) where membrane potential is hyperpolarized. This reduction coincided with diminished Ca^2+^ wave generation, asynchronous events of Ca^2+^ release from the sarcoplasmic reticulum, insensitive to L-type Ca^2+^ channel blockade (Nifedipine, 0.3 µM). Proximity ligation assay (PLA) confirmed IP_3_R1/Ca_V_3.1 close physical association. IP_3_R blockade (2-APB, 50 µM or xestospongin C, 3 µM) in nifedipine-treated C57BL/6 arteries rendered a Ca_V_3.1^−/−^ contractile phenotype. Findings indicate that Ca^2+^ influx through Ca_V_3.1 contributes to myogenic tone at hyperpolarized voltages through Ca^2+^-induced Ca^2+^ release tied to the sarcoplasmic reticulum. This study helps establish Ca_V_3.1 as a potential therapeutic target to control blood pressure.

## Introduction

Smooth muscle cells in the arterial wall actively contract to intravascular pressure, maintaining organ blood flow under dynamic conditions^1,2^. This “myogenic response” was first described by Bayliss^3^ and is intimately tied to arterial depolarization, the activation of voltage-gated Ca^2+^ channels and the concomitant rise in cytosolic Ca^2+^ concentration ([Ca^2+^]_i_), which complexes with calmodulin driving myosin light chain phosphorylation^4^. Three subclasses of voltage-gated Ca^2+^ channels (Ca_V_1–3), are encoded in the mammalian genome and each displays unique voltage-dependent properties^5^. In arterial smooth muscle, Ca_V_1.2 (L-type) Ca^2+^ channels are principally responsible for extracellular Ca^2+^ entry and their blockade is notable for attenuating a range of constrictor responses, including those induced by pressure^6^.

L-type Ca^2+^ channels are classified as high-voltage-activated and dominate the setting of smooth muscle [Ca^2+^]_i_ when intravascular pressure is elevated and arteries depolarized^7^. Their activity, however, markedly drops with hyperpolarization as pressure is reduced or when endothelial cell K^+^ channels are activated by selected agents^8^. As [Ca^2+^]_i_ remains a determinant of tone, even in the hyperpolarized state, it follows that other Ca^2+^ channels, ones with a leftward voltage profile, should be expressed in vascular smooth muscle^6^. T-type Ca^2+^ channels display activation/inactivation properties decidedly more negative to their L-type counterparts^9,10^. Two subtypes (Ca_V_3.1 and Ca_V_3.2) are expressed in vascular smooth muscle, the latter linked to the activation of large-conductance Ca^2+^-activated K^+^ (BK) channels and a negative feedback response limiting arterial constriction^10^. This leaves Ca_V_3.1 as to enabling myogenic tone at hyperpolarized voltages^9,11,12^, presumptively through a mechanism where Ca^2+^ influx directly contributes to the cytosolic Ca^2+^ pool or indirectly triggers sarcoplasmic reticulum Ca^2+^ release in the form of asynchronous Ca^2+^ waves^13–15^.

This study explored whether and by what mechanisms Ca_V_3.1 channels enable myogenic tone development in mouse mesenteric arteries. This work entailed the use of C57BL/6 wild type and Ca_V_3.1^−/−^ mice, and the integrated use of cellular (patch-clamp electrophysiology, immunofluorescence, and PLA), tissue (pressure myography and rapid Ca^2+^ imaging) and whole animal (metabolic caging and blood pressure) techniques. Initial assays confirmed the absence of Ca_V_3.1 in mesenteric arterial smooth muscle of knockout animals. This absence aligned with a drop in systemic blood pressure and reduced myogenic tone at lower pressures compared to controls. Subsequent experiments revealed that Ca^2+^ wave generation was attenuated in Ca_V_3.1^−/−^ arteries as this channel no longer resided near IP_3_R1 and that IP_3_R blockade in C57BL/6 arteries produced a Ca_V_3.1^−/−^ phenotype. These findings highlight a role for Ca_V_3.1 in myogenic tone development and hemodynamic control through the triggering of sarcoplasmic reticulum Ca^2+^ waves. They additionally reveal the potential therapeutic value of Ca_V_3.1 in the control of hypertension.

## Materials and methods

### Animal and tissue preparation

Animal procedures were approved by the animal care committee at the University of Western Ontario ensuring compliance with federal and provincial standards and under consideration of the ARRIVE guidelines. Male C57BL/6 (wild type; Jackson labs) or Ca_V_3.1^−/−^ (in-house colony) mice (16–20 weeks of age) were humanely euthanized via CO_2_ asphyxiation. The mesentery was removed rapidly and placed in cold PBS solution (pH 7.4) containing (in mM): 138 NaCl, 3 KCl, 10 Na_2_HPO_4_, 2 NaH_2_PO_4_, 5 glucose, 0.1 CaCl_2_, and 0.1 MgSO_4_. Third and fourth-order mesenteric were isolated and cut into 2–3 mm segments and transferred to fresh cold PBS.

### Polymerase chain reaction

Ear tissue was collected from C57BL/6 and Ca_V_3.1^−/−^ mice (n = 3) and DNA was extracted using the QIAamp Fast DNA Tissue Kit (QIAGEN). 200 ng of DNA was amplified via polymerase chain reaction (PCR) using previously published Ca_V_3.1 primers: C57BL/6 F, 5ʹ-ATACGTGGTTCGAGCGAGTC-3ʹ; WT R, 5ʹ-CGAAGGCCTGACGTAGAAAG-3ʹ; Ca_V_3.1^−/−^ R, 5ʹ-CTGACTAGGGGAGGAGTAGAAG-3ʹ^16^. Gel electrophoresis was performed on PCR products at 95 V for 1 h on a 1.5% agarose gel. Gel was then imaged using the Bio-Rad ChemiDoc™ MP Imaging System and Image Lab 6.1 software (Bio-Rad).

### Isolation of mesenteric arterial smooth muscle cells

Third and fourth-order mesenteric arteries were placed in an isolation medium (37 °C, 10 min) containing (in mM): 60 NaCl, 80 Na-glutamate, 5 KCl, 2 MgCl_2_, 10 glucose and 10 HEPES with 1 mg/mL bovine serum albumin (BSA, pH 7.4). Vessels were then exposed to a two-step digestion process that began with 14-min incubation (37 °C) in media containing 0.5 mg/mL papain and 1.5 mg/mL dithioerythritol, followed by 10-min incubation in media containing 100 μM Ca^2+^, and collagenases type H (0.7 mg/mL) and type F (0.4 mg/mL). Following incubation, tissues were washed repeatedly with ice-cold isolation medium and triturated with a fire-polished pipette. Liberated cells were stored in ice-cold isolation medium for use the same day.

### Immunohistochemistry

Ca_V_3.1 expression was assessed in mesenteric arterial smooth muscle cells isolated from C57BL/6 and Ca_V_3.1^−/−^ mice. Briefly, isolated cells were fixed onto a microscope cover glass in PBS (pH 7.4) containing 4% paraformaldehyde and 0.2% Tween 20. Fixed cells were blocked (1 h, 22 °C) with a quench solution (PBS supplemented with 3% donkey serum and 0.2% Tween 20) and subsequently incubated overnight (4 °C, humidified chamber) with rabbit anti-Ca_V_3.1 primary antibody diluted in quench solution (1:100). In the following morning, cells were washed 3× in PBS-0.2% Tween 20 and then incubated (1 h, 22 °C) in a PBS-0.2% Tween 20 buffer containing Alexa Fluor 488 donkey anti-rabbit IgG-secondary antibody (1:200). After further washing, isolated cells and whole-mount preparations were mounted with Prolong Diamond Antifade Mountant with DAPI. Immunofluorescence was detected through a 63× oil immersion lens coupled to a Leica-TCS SP8 confocal microscope equipped with the appropriate filter sets. Smooth muscle cells isolated from C57BL/6 cerebral arteries were used as Ca_V_3.1 positive controls. Secondary antibody controls were performed and were negative for nonselective labelling.

### Electrophysiological recordings

Conventional patch-clamp electrophysiology was utilized to measure voltage-gated Ca^2+^ currents in smooth muscle cells isolated from mesenteric arteries. Cell averaged capacitance was 12–18 pF. Recording electrodes (pipette resistance, 5–8 MΩ) were fashioned from borosilicate glass using a micropipette puller (Narishige PP-830, Tokyo, Japan) and backfilled with pipette solution containing (in mM): 135 CsCl, 5Mg-ATP, 10 HEPES, and 10 EGTA (pH 7.2). To attain a whole-cell configuration, the pipette was lowered onto a cell while applying negative pressure to rupture the membrane and garner intracellular access. Cells were voltage clamped (holding potential: − 60 mV) and subjected to − 90 mV followed by 10 mV voltage steps (300 ms) starting from − 50 to 40 mV in a bath solution consisting of (mM): 110 NaCl, 1 CsCl, 1.2 MgCl_2_, 10 glucose, and 10 HEPES plus 10 BaCl_2_ (charge carrier). Delineation of vascular voltage-gated Ca^2+^ channels was performed by introducing 200 nM nifedipine to block L-type channels, followed by 50 µM Ni^2+^ to selectively block Ca_V_3.2 channels without affecting Ca_V_3.1. Currents were recorded using an Axopatch 200B patch-clamp amplifier (Molecular Devices, Sunnyvale, CA) at room temperature (~ 22 °C). Data were filtered at 1 kHz, digitized at 5 kHz, and stored on a computer for offline analysis with Clampfit 10.3 software (Molecular Devices, Sunnyvale, CA). Current/voltage relationships were plotted as peak current density (pA/pF) at the different voltage steps.

### Indirect calorimetry, activity, and inactivity

Comprehensive Lab Animal Monitoring System (CLAMS) interface using Oxymax software (Columbus Instruments, Columbus, OH) was utilized to measure the differences in O_2_ consumption and CO_2_ production, the cumulative amount of food and water consumed, respiratory exchange rate, activity (number of infrared beam breaks), and sleep epochs were measured in C57BL/6 and Ca_V_3.1^−/−^ mice. Chambers were kept at 24 ± 1 °C with airflow of 0.5 L/min, and animals had ad libitum access to food and water. Metabolic parameters were recorded every 10 min for 48 h. Data from the same 12-h interval for each mouse was selected to standardize the data processing.

### Blood pressure and heart rate assessment

Blood pressure measurements were performed on awake C57BL/6 and Ca_V_3.1^−/−^ mice using the non-invasive CODA tail-cuff system (Kent Scientific, CT), as described, and following recommendations of the Subcommittee of Professional and Public Education of the American Heart Association Council on High Blood Pressure Research^17,18^. To minimize anxiety, the animals were properly acclimatized in advance, and a heating platform was used to maintain body temperature. Mice (*n* = 5) were subjected to 25-min recordings daily for one week, and the weekly averages were recorded.

### Pressure myography

Isolated mesenteric arteries were cannulated in an arteriograph and superfused with physiological salt solution (PSS; 5% CO_2_, balance air) at 37 °C containing (in mM): 119 NaCl, 4.7 KCl, 1.7 KH_2_PO_4_, 1.2 MgSO_4_, 1.6 CaCl_2_, 10 glucose, and 20 NaHCO_3_. To limit the influence of endothelial receptors, air bubbles were passed through the vessel lumen (1 min). Arterial diameters were monitored using an automated edge detection system (IonOptix, MA) and a 10× objective. Arteries were equilibrated at 15 mmHg, and contractile responsiveness was assessed by a brief (≈ 10 s) application of 60 mM KCl. After equilibration, intraluminal pressure was elevated from 20 to 100 mmHg in 20 mmHg increments for 10 min each, and arterial diameters were monitored in Ca^2+^ PSS (control), and in the presence of 0.3 µM nifedipine (L-type Ca^2+^ Channel blocker) alone or with 50 µM 2-APB or 3 µM xestospongin C (IP_3_R blockers) or 150 nM kurtoxin (Ca_V_3.x blocker). A final passive diameter assessment was conducted in Ca^2+^-free + 2 mM EGTA. Arteries that did not respond to superfused KCl (60 mM) or were insensitive or hypersensitive to pressure were excluded from experimentation. Percentage of maximum tone and incremental distensibility were calculated as follows:

$$\text{\% Maximum tone}=\frac{\text{Max diameter }-\text{ Diameter under control or treated conditions}}{\text{Max diameter}} \times 100,$$

$$\% \text{Incremental distensibility}=\frac{\mathrm{\Delta\, Diameter}}{\mathrm{\Delta\, Pressure}} \times 100.$$

### Agonist-induced constriction

Endothelium-denuded mesenteric arteries were cannulated in a pressure myograph as explained above. Following the high potassium challenge, arteries were equilibrated at 60 mmHg, then subjected to the administration of phenylephrine (PE) into the bath solution. Increasing concentrations of PE (in M) 10^–7^, 3 × 10^–7^, 10^–6^, 3 × 10^–6^, 10^–5^, and 3 × 10^–5^ were superfused into the bath containing PSS in the absence (control) or presence of 0.3 µM nifedipine. Changes in diameter in response to each concentration were recorded and percentage of maximum constriction was calculated as follows:

$$\text{\% Maximum constriction}=\frac{D0-D}{D0-Dm} \times 100$$

where $D$ is the external diameter after each agonist concentration application, $D0$ is the external diameter in Ca^2+^ PSS, and $Dm$ is the external diameter after the highest concentration of agonist under control condition.

### Calcium imaging

Freshly isolated arteries were incubated with the Ca^2+^ indicator Fluo-8 and placed on the stage of a Nikon swept-field confocal microscope with enclosed Agilent 3B laser attached to Andor camera (iXon Ultra). Fluo-8 working solution (19.1 µM) was freshly prepared by dissolving 5 μL of stock solution (1.91 mM) in 5 μL pluronic acid plus 490 μL HBSS buffer consisting of (in mM): 134 NaCl, 6 KCl, 1 MgCl_2_, 2 CaCl_2_ 10 HEPES, and 10 Glucose (pH 7.4). Incubation was done for 75 min at 37 °C in the dark. Vessels were then cannulated and equilibrated at an intraluminal pressure of 15 mmHg for 15 min in Ca^2+^ PSS solution. Intraluminal pressure was then raised to 60 mmHg, and Ca^2+^ waves were recorded in the presence and absence of 0.3 µM nifedipine (± 150 nM kurtoxin) or 50 µM 2-APB or 3 µM xestospongin C. Fluo-8-loaded arteries were excited at 488 nm and emission spectra at 510 nm viewed through a 60× water immersion objective (1.2 WI) and were monitored and analysed using Nikon NIS Elements software (AR 4.20.01). To limit laser-induced tissue injury, image acquisition was set to 45 s at 5 fps. A series of regions of interest (1 × 1 µm), created within the analysis software, was placed on 10 successive cells that were in sharp focus using the first visibly loaded smooth muscle cell as a starting point. A Ca^2+^ wave was defined as local fractional fluorescence ($\text{F}/{\text{F}}_{0}$) increase above the noise level of 1.1, which spans the whole cell and lasts for at least 1 s. Ca^2+^ waves were assessed by the number of firing cells in an array of the 10 adjacent cells and the frequency of Ca^2+^ waves propagation per cell per minute.

### Proximity ligation assay

To test the spatial proximity of Ca_V_3.1 and IP_3_R, the Duolink in situ PLA detection kit was employed as previously described^19^. Briefly, freshly isolated mesenteric arterial smooth muscle cells underwent successive steps of fixation (10% paraformaldehyde in PBS, 15 min), permeabilization (0.2% Tween 20 in PBS, 15 min) and blocking (Duolink blocking solution, 1 h). Cells were then washed with PBS then incubated with primary antibodies (anti-Ca_V_3.1, anti-IP_3_R1) in Duolink antibody diluent solution at 4 °C overnight. Cells were then incubated with secondary Duolink PLA PLUS and MINUS probes for 1 h at 37 °C. If target proteins are within 40 nm of each other, synthetic oligonucleotides attached to the probes hybridize enabling their subsequent amplification and binding to complementary fluorescent oligonucleotide sequences, detected using Leica-TCS SP8 confocal microscope.

### Statistical analysis

Data are expressed as means ± SD, and *n* indicates the number of cells, arteries, or animals. Power analysis was performed a priori to assess the sample size sufficient for obtaining statistical significance. No more than 1 experiment was performed on cells/tissues from any given animal. Where appropriate, paired/unpaired t-tests or two‐way analysis of variance (ANOVA) were performed to ascertain significant differences in mean values to a given condition/treatment. P values ≤ 0.05 were considered statistically significant.

### Solutions and chemicals

Fluo-8 was acquired from Abcam. Primary antibodies against Ca_V_3.1 and IP_3_R1 were purchased from NovusBio and Alomone Laboratories, respectively. PCR kit was obtained from Qiagen. Secondary antibody, Alexa Fluor 488 Donkey Anti-Rabbit IgG (H+L), and 2-APB were obtained from ThermoFisher. Duolink PLA detection kits, nifedipine, PE hydrochloride, DAPI, donkey serum kurtoxin, and all other chemicals were obtained from Sigma-Aldrich unless stated otherwise. In cases where DMSO was used as a solvent, the maximal DMSO concentration after application did not exceed 0.5%. Please see the Major Resources Table in the Supplemental Materials.

## Results

### Characterization of Ca_V_3.1^−/−^ genotype and phenotype

**Figure 1:**
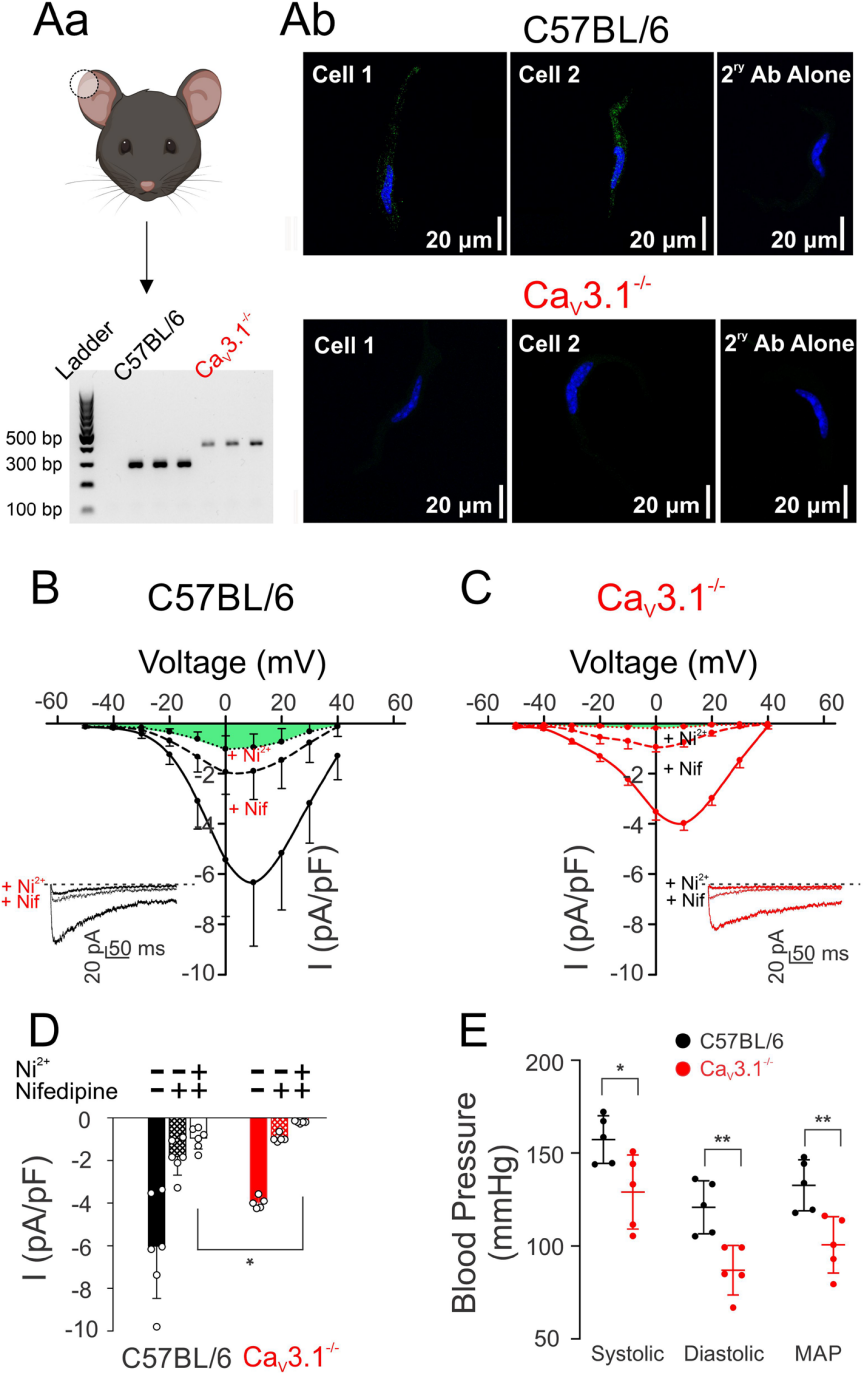
Absence of Ca_V_3.1 expression and current in SMCs isolated from mesenteric arteries of Ca_V_3.1^−/−^ mice, and lower arterial blood pressure indices in Ca_V_3.1^−/−^ mice compared to C57BL/6. **(Aa)** Polymerase chain reaction of Cacna1g gene (Ca_V_3.1). DNA was extracted from ear notches (C57BL/6 and Ca_V_3.1^−/−^ mice) and amplified; the different product sizes confirm the gene modification leading to functional knockout. Illustration created with BioRender.com. **(Ab)** Ca_V_3.1 (green) in cerebral arterial myocytes from control mice with nuclei stained with DAPI (blue) detected with immunohistochemistry. This signal was absent in Ca_V_3.1^−/−^ mice. Secondary antibody controls were negative for nonselective labelling (*n* = 4 cells pooled from 4 animals/group). **(B)** Averaged Ca_V_ currents were assessed by whole-cell patch clamp in C57BL/6 cells showing a residual current remaining (highlighted green) after blocking L-type and Ca_V_3.2 currents by nifedipine and Ni^2+^, respectively. **(C)** Recordings of whole-cell Ca_V_ currents in Ca_V_3.1^−/−^ cells showing no residual current after nifedipine and Ni^2+^ treatment. **(D)** Peak current (I) plots of whole-cell Ba^2+^ (10 mmol/L) current before and after the application of nifedipine to C57BL/6 and Ca_V_3.1^−/−^ smooth muscle cells. *n* = 9 SMCs from 6 mice in control group and *n* = 9 SMCs from 8 mice in knockout group. (* *P* = 0.013, unpaired *t* test). **(E)** Systolic, diastolic, and mean arterial pressure (mmHg) of Ca_V_3.1^−/−^ and C57BL/6 mice were measured using the CODA6 tail-cuff system. 25-min recordings daily for one week were performed on both groups (*n* = 5). (Systolic: *P = 0.028, Diastolic: **P = 0.005, MAP: **P = 0.008, unpaired *t* test).

Genetic deletion of Ca_V_3.1 channels was confirmed by PCR, immunohistochemical analysis and conventional whole-cell patch-clamp electrophysiology. In detail, PCR amplification of C57BL/6 and Cav3.1^−/−^ mouse DNA with Ca_V_3.1 primers resulted in different sized PCR products. C57BL/6 mice had a PCR product of 288 bp corresponding to the wild type allele, while the PCR product for Ca_V_3.1^−/−^ mice was seen at 385 bp (Fig. 1Aa). Figure 1Ab shows Ca_V_3.1 protein expression is punctate in smooth muscle cells isolated from C57BL/6 but not Ca_V_3.1^−/−^ mesenteric arteries (n = 4 mice per group). This analysis aligned with whole-cell electrophysiology, which noted dampened Ca_V_3.1 activity in smooth muscle cells from Ca_V_3.1^−/−^ mice relative to C57BL/6 controls (*P* = 0.013). Note, Ca_V_3.1 activity was measured by first monitoring the total inward Ba^2+^ current, the collective sum of Ca_V_1.2, Ca_V_3.1, and Ca_V_3.2 currents^20^. Based on past studies, nifedipine and Ni^2+^ were then applied to abolish Ca_V_1.2 (L-type) and Ca_V_3.2 (T-type) activities, respectively, and the residual current was then assigned to Ca_V_3.1^21,22^. The current–voltage relationship of each Ca^2+^ channel is illustrated in Fig. 1B,C (Ca_V_3.1 current in green), with peak current (at + 10 mV) summarized in Fig. 1D. Recordings were attained from mesenteric smooth muscle cells (9 cells per group) isolated from 6 C57BL/6 and 8 Ca_V_3.1^−/−^ mice.

### Metabolic and blood pressure measurements in C57BL/6 and Cav3.1^−/−^ mice

Metabolic caging assessed O_2_ consumption, CO_2_ production, the respiratory exchange rate, along with cumulative food and water consumed during normal activity. No significant difference was observed among C57BL/6 and Ca_V_3.1^−/−^ mice (Table 1) in these parameters. However, Ca_V_3.1^−/−^ mice displayed a disrupted night-time sleeping pattern and were modestly but significantly heavier than the C57BL/6 controls. Subsequent tail-cuff measurements revealed that systolic, diastolic, and consequently mean arterial pressures were reduced in Ca_V_3.1^−/−^ mice compared to C57BL/6 mice (Fig. 1E).

**Table 1:** There are no discernible metabolic differences among strains, except sleep time and weight.

Metabolic parameters	Condition	C57BL/6	Ca_V_3.1^−/−^
VO_2_ (mL/kg h)	Light	3186 ± 216	2902 ± 57.7
	Dark	3851 ± 209	3644 ± 57.5
VCO_2_ (mL/kg h)	Light	3078 ± 190	2716 ± 55.4
	Dark	3865 ± 204	3624 ± 50.4
RER (VCO_2_/VO_2_)	Light	0.968 ± 0.01	0.935 ± 0.01
	Dark	1.004 ± 0	0.994 ± 0.01
Food consumption (mg)	Light	1.459 ± 0.14	1.67 ± 0.21
	Dark	2.998 ± 0.28	2.832 ± 0.42
Water consumption (mL)	Light	1.09 ± 0.14	1.61 ± 0.27
	Dark	3.273 ± 0.19	4.063 ± 0.4
Sleep time (min)	Light	412 ± 15.1	420 ± 13.9
	Dark	217.2 ± 12.1	161.7 ± 13.4
Weight (g)	N/A	24.4 ± 1.76	28.25 ± 0.65
Heart rate (bpm)	N/A	690.9 ± 25.6	679.9 ± 27.7

C57BL/6 and Ca_V_3.1^−/−^ mice were placed into individual CLAMS metabolic chambers for 48 h. Metabolic parameters including VO_2_, VCO_2_, respiratory exchange rate (RER), food and water consumption, and sleep times, in both light and dark conditions were measured (*n* = 6 for each group). Data were presented as means ± SE and compared using unpaired two-tailed *t*-tests. Numbers in red indicate a significant difference of P < 0.05.

### Ca_V_3.1 channels contribute to the myogenic response

**Figure 2:**
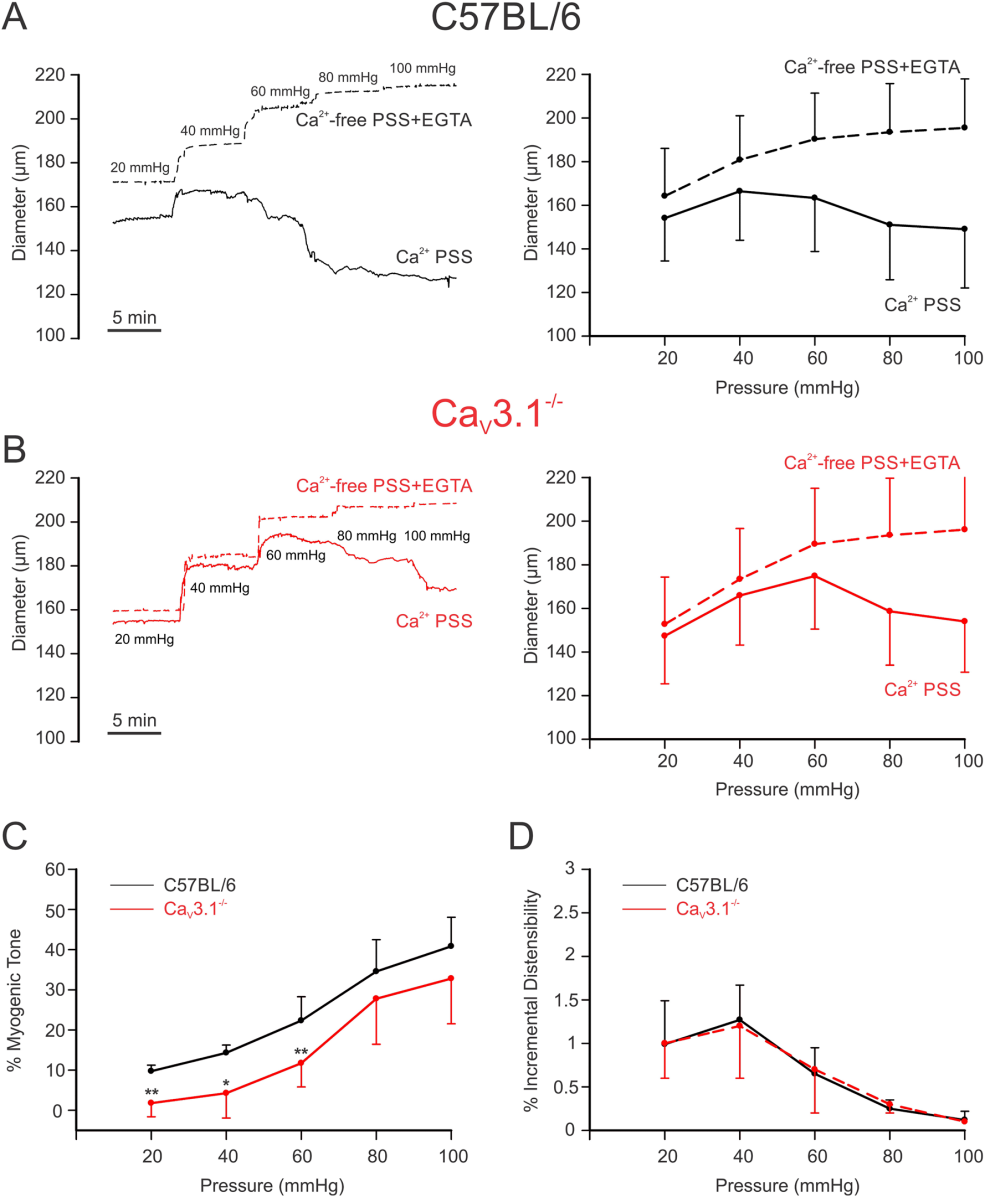
Arteries from Ca_V_3.1^−/−^ mice develop less myogenic tone at low intraluminal pressures. Isolated mesenteric arteries from Ca_V_3.1^−/−^ and C57BL/6 mice underwent a pressure curve in two conditions: PSS containing Ca^2+^ and Ca^2+^ free PSS + 2 mM EGTA, a Ca^2+^-chelating agent. **(A,B)** Representative trace and summary data of changes in diameter in response to pressure curve (20–100 mmHg) in C57BL/6 and Ca_V_3.1^−/−^. **(C)** Summary data shows arteries from Ca_V_3.1^−/−^ mice had lower myogenic tone in the pressure range from 20–60. (20 mmHg: **P = 0.002, 40 mmHg: *P = 0.014, 60 mmHg: **P = 0.008, 80 mmHg: *P* = 0.188, 100 mmHg: *P* = 0.108, unpaired *t* test). **(D)** Summary data of incremental distensibility shows no difference between groups. (*n* = 6 arteries from 6 animals for each experiment). (20 mmHg: *P* = 0.945, 40 mmHg: *P* = 0.651, 60 mmHg: *P* = 0.571, 80 mmHg: *P* = 0.793, 100 mmHg: *P* = 0.245, unpaired *t* test).

**Figure 3:**
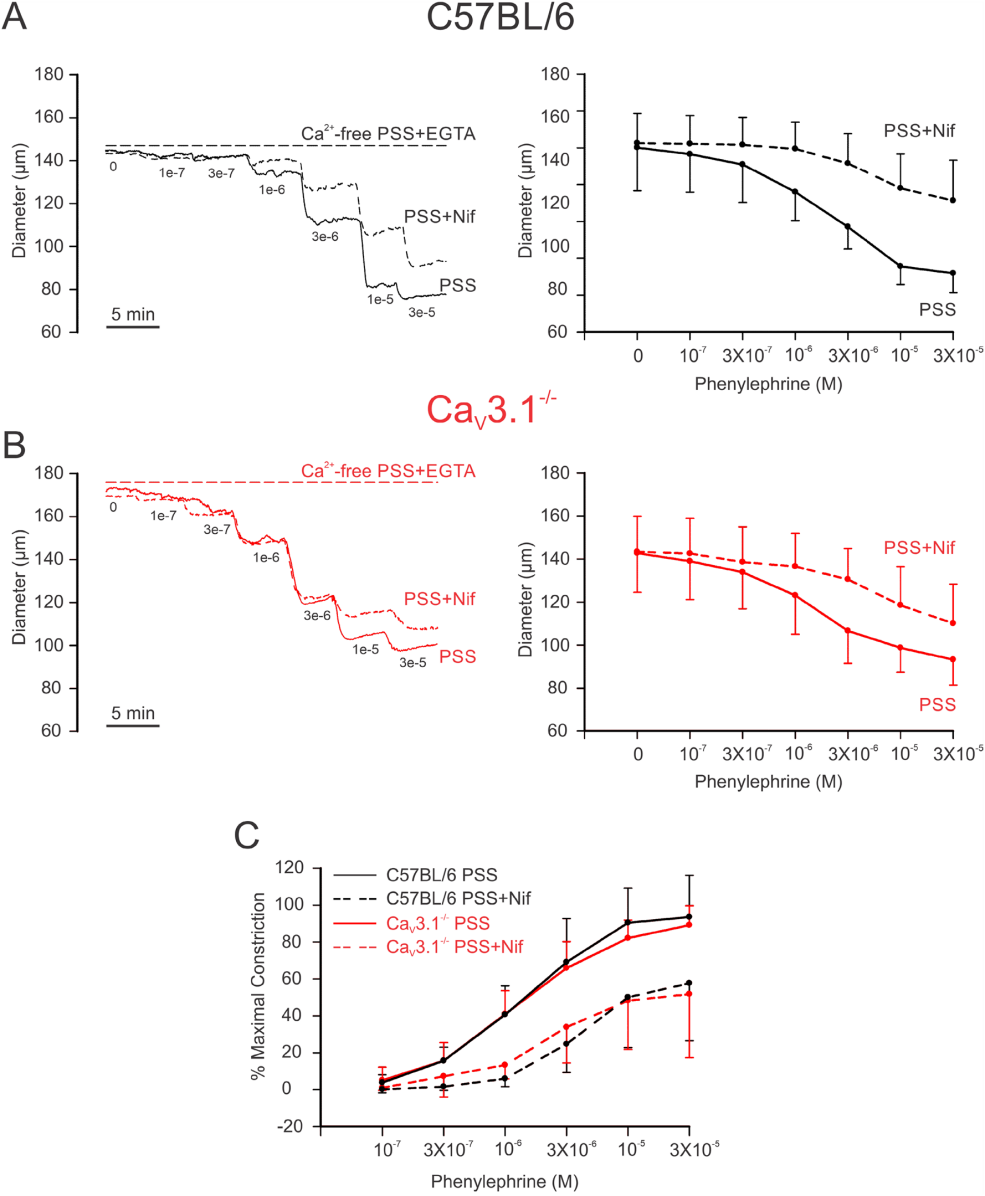
Ca_V_3.1 deletion has no impact on phenylephrine-induced constriction. Increasing concentrations of phenylephrine were applied onto pressurized arteries isolated from C57BL/6 and Ca_V_3.1^−/−^ mice in the presence and absence of nifedipine (L-type Ca^2+^ channel blocker). Experiments were conducted at an intraluminal pressure of 60 mmHg. **(A,B)** Representative traces (Left) and summary data (Right) of changes in diameter in response to phenylephrine showing a decrease in constriction in nifedipine-treated vessels from both strains. **(C)** %Maximal phenylephrine-induced constriction relative to KCl-induced constriction shows no significant difference in agonist-induced constriction between C57BL/6 and Ca_V_3.1^−/−^ mice. (*n* = 6 arteries from 6 animals). *P* values for increasing PE concentrations in PSS: 0.529, 0.790, 0.763, 0.957, 0.554, 0.719 and in PSS + nifedipine: 0.565, 0.343, 0.074, 0.396, 0.925, 0.837 (Paired *t* test).

Mesenteric arteries from C57BL/6 and Ca_V_3.1^−/−^ mice were mounted in a myograph and exposed to increasing intraluminal pressures (20 to 100 mmHg) in physiological saline solutions, with Ca^2+^ and Ca^2+^-free + 2 mM EGTA. Traces and summative data are presented in Fig. 2A–C, and findings reveal that Ca_V_3.1^−/−^ arteries displayed reduced myogenic tone compared to C57BL/6 controls, a trend that was statistically significant at lower intraluminal pressures (20 mmHg: *P* = 0.002, 40 mmHg: *P* = 0.014, 60 mmHg: *P* = 0.008, 80 mmHg: *P* = 0.188, 100 mmHg: *P* = 0.108, unpaired *t* test). Arterial distensibility, a surrogate of vessel stiffness and defined as the percentage change in passive arteriolar diameter per change in intravascular pressure^23^, was comparable among the two groups of arteries (Fig. 2D). Control experiments using PE as a vasoconstrictor noted a comparable vasomotor tone among C57BL/6 and Ca_V_3.1^−/−^ arteries across a full concentration range (Fig. 3A,B). This statement applies equally to tone generated in the absence and presence of nifedipine, except at the higher agonist concentrations where the L-type Ca^2+^ channel blocker initially appeared to be less impactful in Ca_V_3.1^−/−^ arteries (Fig. 3A,B). Note, however, when this data was normalized to the % maximal constriction, the nifedipine-sensitive and insensitive components of agonist-induced constriction were comparable among the two groups of arteries (Fig. 3C).

### Ca_V_3.1 enable myogenic tone by facilitating Ca^2+^ wave generation

**Figure 4:**
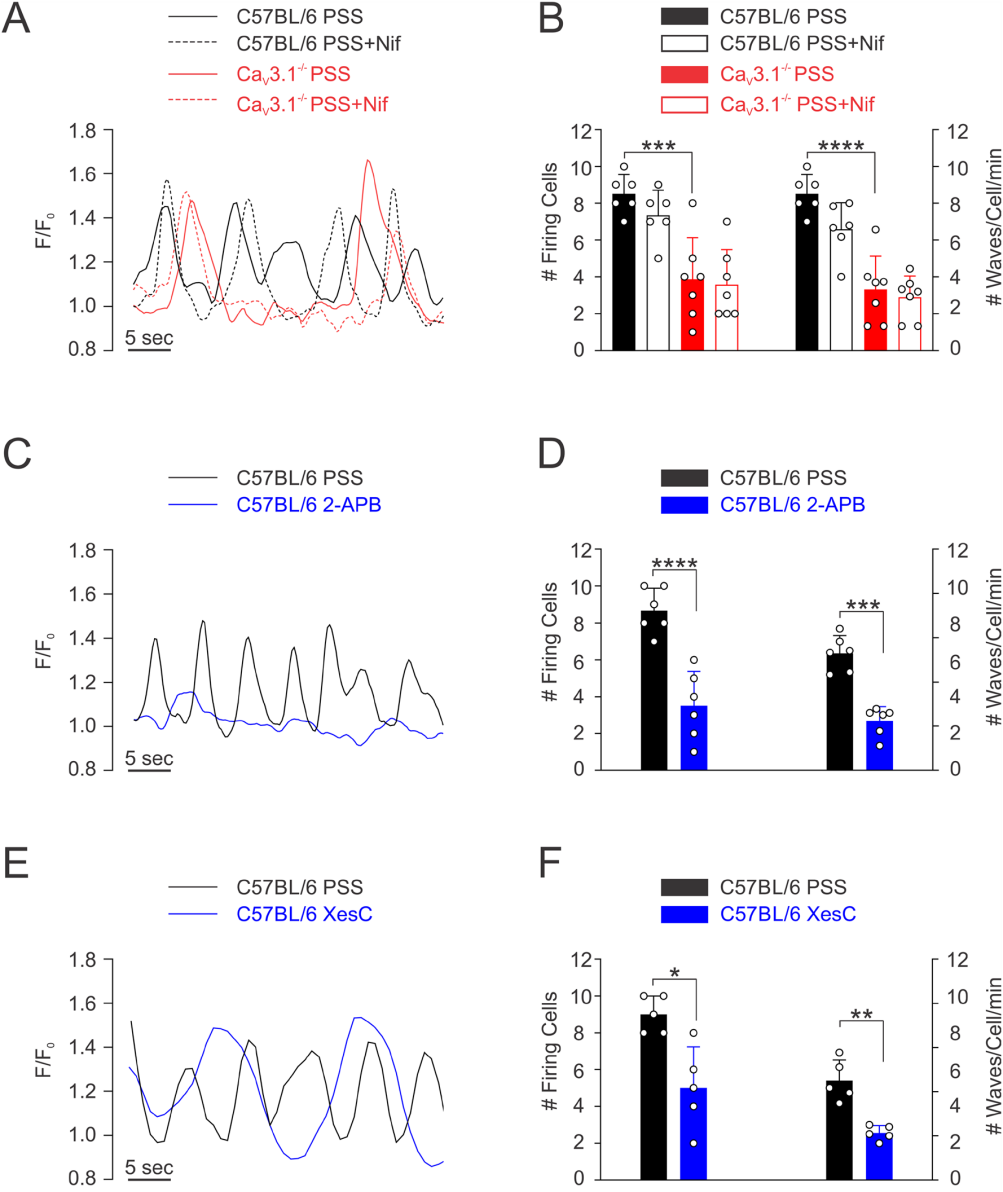
Functional roles of Ca_V_3.1 and IP_3_Rs in Ca^2+^ waves generation. Rapid Ca^2+^ imaging was performed on Fluo-8-loaded arteries from Ca_V_3.1^−/−^ and C57BL/6 mice at an intraluminal pressure of 60 mmHg. **(A)** Representative traces from C57BL/6 and Ca_V_3.1^−/−^ mesenteric arteries with and without nifedipine. **(B)** Summary data (*n* = 6 arteries from 6 mice). Number of cells firing (***P = 0.0002) and firing frequency (***P = 0.0001) were significantly reduced in Ca_V_3.1^−/−^ when compared to C57BL/6. Nifedipine did not impact the number of cells firing (C57BL/6: *P* = 0.485, Ca_V_3.1^−/−^ *P* = 0.980) or the firing frequency (C57BL/6: *P* = 0.093, Ca_V_3.1^−/−^ *P* = 0.925) in either strain. *P* values were calculated using 2-way ANOVA. **(C)** Representative traces from C57BL/6 mesenteric arteries with and without 2-APB. **(D)** Summary data (*n* = 6 arteries from 6 mice). 2-APB (IP_3_R inhibitor) decreased the number of cells firing and their firing frequency (****P < 0.0001 and ***P = 0.0002, respectively, paired *t* test) in mesenteric arteries from C57BL/6 mice. **(E)** Representative traces from C57BL/6 mesenteric arteries with and without xestospongin C. **(F)** Summary data (*n* = 5 arteries from 5 mice). xestospongin C (IP_3_R inhibitor) decreased the number of cells firing and their firing frequency (*P < 0.011 and **P = 0.002, respectively, paired *t* test) in mesenteric arteries from C57BL/6 mice. *F* fluorescence intensity, *F*_*o*_ baseline fluorescence.

**Figure 5:**
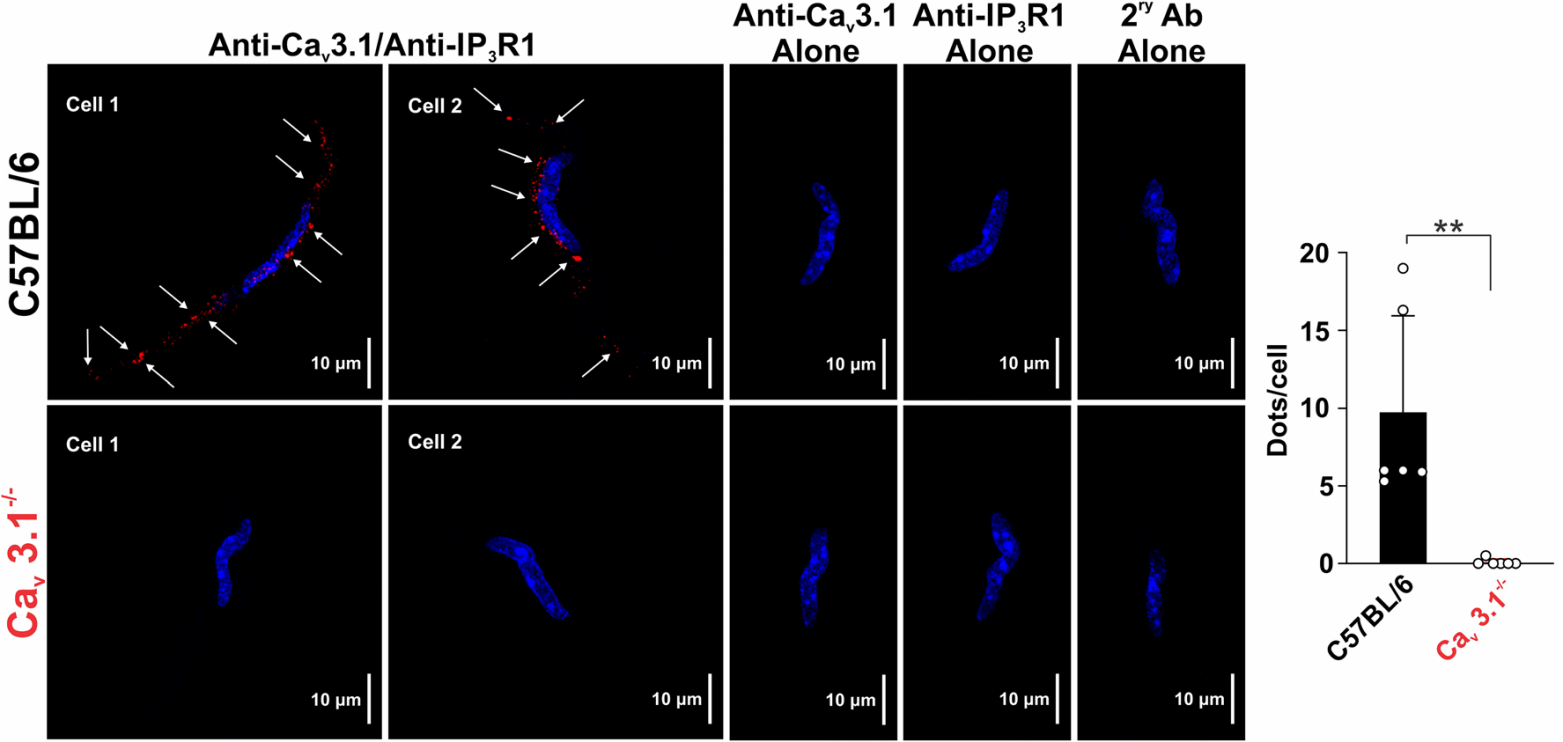
Ca_V_3.1 channels colocalize with IP_3_Rs. Proximity ligation assay was employed using isolated mesenteric arterial SMCs from C57BL/6 (*n* = 35 cells from 6 animals) and Ca_V_3.1^−/−^ (*n* = 36 cells from 6 animals) mice to determine the close association (< 40 nm) of Ca_V_3.1 and IP_3_R1 proteins (red, denoted by white arrows). Nuclei were stained with DAPI (Blue). Control experiments used only one primary antibody or no primary antibody. Note, dots were averaged across cells within each animal, and represented as a data point in the bar graph to facilitate statistical comparison. **P = 0.0033.

To assess whether Ca^2+^ flux through Ca_V_3.1 triggers Ca^2+^ wave generation, mesenteric arteries from C57BL/6 and Ca_V_3.1^−/−^ mice were loaded with Fluo-8, and rapid Ca^2+^ imaging was assessed by swept field confocal microscopy. Ca^2+^ waves in C57BL/6 mice were readily observed in 80% of smooth muscle cells (8 of 10 per vessel) at a frequency of 9 waves/cell/min, each with a duration of 3–4 s (Fig. 4A,B). Similar to rat vessels, nifedipine application had little discernible effect on Ca^2+^ wave generation^24^. The deletion of Ca_V_3.1 markedly reduced the number of firing smooth muscle cells (*P* = 0.0002) along with firing frequency (*P* < 0.0001) by 55% and 65%, respectively (Fig. 4A,B); the Ca^2+^ waves that remained were insensitive to nifedipine. Control experiments in C57BL/6 mesenteric arteries (Fig. 4C,D) subsequently confirmed that 2-APB, a blocker of IP_3_Rs, notably attenuated the number of firing cells (*P* < 0.0001) and Ca^2+^ wave frequency (*P* = 0.0002) by 80% and 75%, respectively. Owing to the non-selective nature of 2-APB, and its reported inhibition of store-operated Ca^2+^ entry, the previous control experiments were repeated in the presence of xestospongin C, a selective IP_3_R blocker. Similar to 2-APB, xestospongin C attenuated the number of firing cells (*P* = 0.011) and Ca^2+^ wave frequency (*P* = 0.002) (Fig. 4E,F). With this functional evidence indicating that Ca^2+^ flux through Ca_V_3.1 triggers IP_3_Rs and the induction of Ca^2+^ waves, the PLA was employed to assess whether these two proteins sat closely to one another. Consistent with Ca_V_3.1 and IP_3_R1 residing within 40 nm of one another, we observed red punctate labelling in smooth muscle cells isolated from C57BL/6 but not Ca_V_3.1 mesenteric arteries (Fig. 5). Controls were performed on cells treated with anti-Ca_V_3.1, anti-IP_3_R1, or secondary antibodies alone, and revealed no evidence of nonspecific binding and false product amplification.

**Figure 6:**
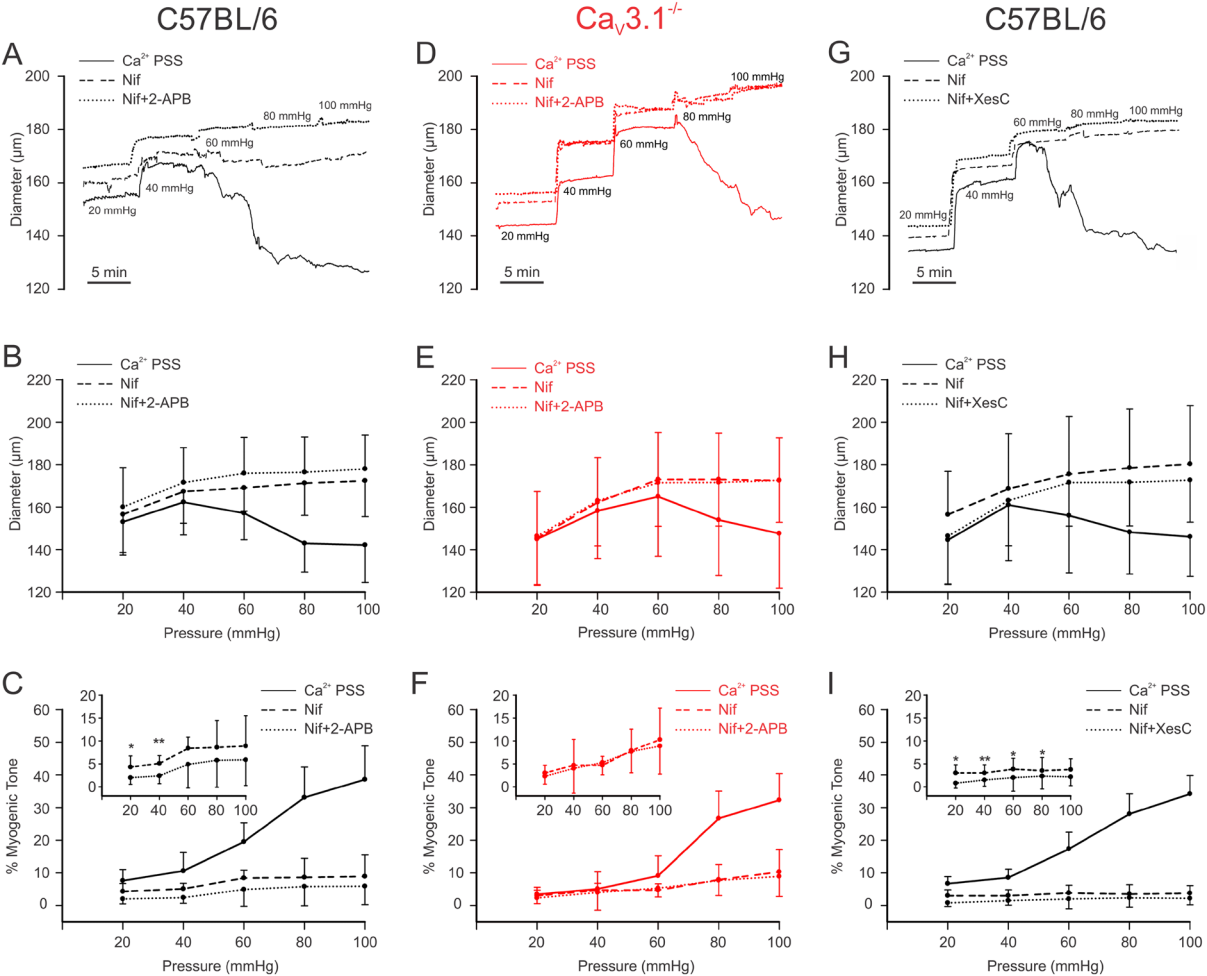
IP_3_R blockade has no impact on myogenic tone development in Cav3.1^−/−^. Mesenteric arteries isolated from C57BL/6 and Ca_V_3.1^−/−^ mice underwent stepwise pressure increases in control conditions (Ca^2+^ PSS), with nifedipine (Ca_V_1.2 blocker) alone and with 2-APB or xestospongin C (IP_3_R blockers). **(A,D,G)** Representative traces and **(B,E,H)** summary data of changes in mesenteric arteriolar diameter in response to pressure steps from C57BL/6 **(A,G)** and Ca_V_3.1^−/−^ mice **(D)** are depicted. In C57BL/6 mice, pressure-induced constriction decreased after nifedipine, 2-APB, and xestospongin C treatment. In Ca_V_3.1^−/−^ mice, the vasomotor response following nifedipine and 2-APB treatment was not different from nifedipine treatment only. **(C,I)** %Myogenic tone was reduced following 2-APB (20 mmHg: *P = 0.013, 40 mmHg: **P = 0.008, 60 mmHg: *P* = 0.124, 80 mmHg: *P* = 0.085, 100 mmHg: *P* = 0.102, paired *t* test) and xestospongin C (20 mmHg: *P = 0.015, 40 mmHg: **P = 0.001, 60 mmHg: *P = 0.02, 80 mmHg: *P = 0.013, 100 mmHg: *P* = 0.092, paired *t* test) treatment at 20–40 pressure range in C57BL/6 mice. **(F)** No changes in myogenic tone were observed following 2-APB treatment in Ca_V_3.1^**−/−**^ mice. (20 mmHg: *P* = 0.182, 40 mmHg: *P* = 0.334, 60 mmHg: *P* = 0.535, 80 mmHg: *P* = 0.899, 100 mmHg: *P* = 0.245, paired *t* test) (*n* = 6 arteries from 6 animals for each experiment).

**Figure 7:**
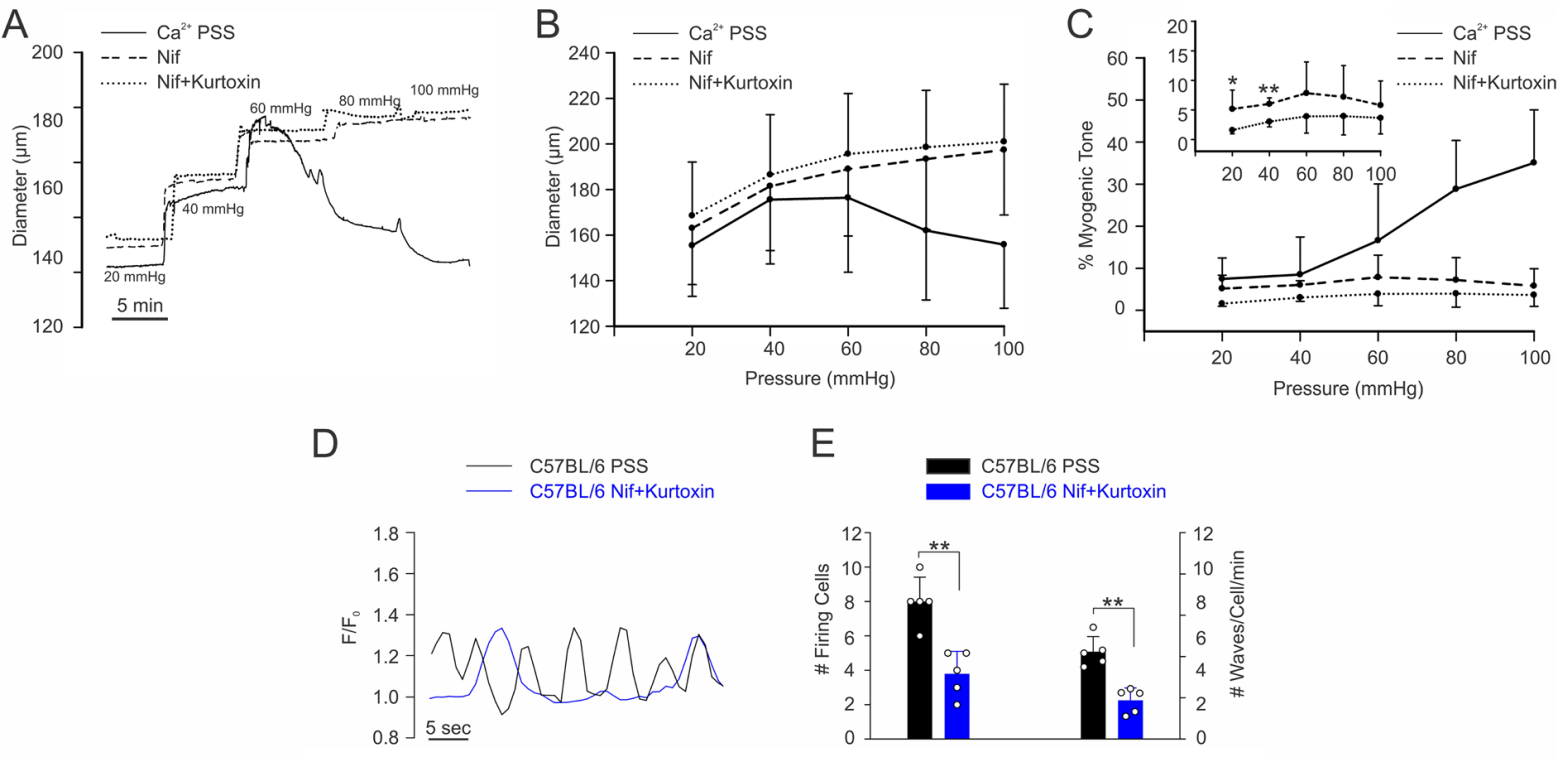
Ca_V_3.x blockade diminishes myogenic tone development and Ca^2+^ wave generation in C57BL/6 mice. Mesenteric arteries isolated from C57BL/6 mice underwent stepwise pressure increases in control conditions (Ca^2+^ PSS), with nifedipine (Ca_V_1.2 blocker) alone and with kurtoxin (150 nM, Ca_V_3.x blocker). **(A)** Representative trace, and summary data of **(B)** arterial diameter and **(C)** % myogenic tone to pressure in C57BL/6 mesenteric vessels. Pressure-induced constriction decreased after nifedipine, and kurtoxin treatment. %Myogenic tone was reduced following kurtoxin (20 mmHg: *P = 0.04, 40 mmHg: **P = 0.008, 60 mmHg: *P* = 0.074, 80 mmHg: *P* = 0.105, 100 mmHg: *P* = 0.116, unpaired *t* test). **(D)** Representative trace of Ca^2+^ wave generation in C57BL/6 mesenteric arteries in the absence and presence of nifedipine + kurtoxin.** (E)** Unlike Fig. 4, where nifedipine had no effect on Ca^2+^ wave generation, summary data (*n* = 5 arteries from 5 mice) reveals that further addition of kurtoxin causes a marked attenuation in the number of cells firing and firing frequency (**P = 0.004 and **P = 0.002, respectively, paired *t* test). *F* fluorescence intensity, *F*_*o*_ baseline fluorescence.

Given the preceding observation, a final set of functional experiments were performed to address the contributory role of IP_3_R-dependent Ca^2+^ waves to pressure-induced constriction. Using mesenteric arteries from C57BL/6 and Ca_V_3.1^−/−^ mice, myogenic tone was examined over a full pressure range in the absence and presence of nifedipine (0.3 µM) ± 2-APB (50 µM). Of particular note, was the nifedipine-resistant tone that was present in C57BL/6 but not Ca_V_3.1^−/−^ arteries, particularly at lower intravascular pressures (Fig. 6A,B,D,E). That tone per se was largely eliminated with the further application of 2-APB (20 mmHg: *P* = 0.013, 40 mmHg: *P* = 0.008) consistent with IP_3_Rs and Ca^2+^ waves playing a role in its genesis (Fig. 6C,I). Note that IP_3_R inhibition in Ca_V_3.1^−/−^ arteries had no discernible effect on nifedipine insensitive tone at any pressure (Fig. 6D–F). In a set of control experiments, xestospongin C (3 µM, a more selective IP_3_R antagonist) was used in place of 2-APB in C57BL/6 and it generated a contractile (Fig. 6G,H, 20 mmHg: *P* = 0.015, 40 mmHg: *P* = 0.001, 60 mmHg: *P* = 0.02, 80 mmHg: *P* = 0.013), and Ca^2+^ wave (Fig. 4E,F) phenotype akin to Ca_V_3.1^−/−^ arteries. In a final set of controls, myogenic tone and Ca^2+^ waves were assessed in C57BL/6 vessels, with and without kurtoxin (Fig. 7) to block Ca_V_3 channels. Kurtoxin reduced myogenic tone at 20 and 40 mmHg when place on top of nifedipine (20 mmHg: *P* = 0.04, 40 mmHg: *P* = 0.008). Also note, whereas nifedipine had no impact on Ca^2+^ wave generation (Fig. 4), kurtoxin + nifedipine had an inhibitory effect (Fig. 7: number of firing cells (*P* = 0.004) and firing frequency (*P* = 0.002)).

## Discussion

Bayliss first described the intrinsic ability of resistance arteries to constrict to a rise in intravascular pressure^3^. This foundational response is now known to set basal tone in key organs and stabilizes organ perfusion as blood pressure fluctuates. Further, this response has been intimately tied to arterial depolarization and the rise in [Ca^2+^]_i_ enabled by graded Ca^2+^ entry principally through L-type Ca^2+^ channels^9^. Vascular L-type Ca^2+^ channels are encoded by the Ca_V_1.2 α_1_ pore-forming subunit whose steady-state voltage-dependent properties are shifted rightward to more depolarized potentials^25^. Recent work has revealed that L-type Ca^2+^ channels are not alone in vascular smooth muscle and that T-type Ca^2+^ channels are also expressed, with Ca_V_3.1 being key to this investigation^9^. Its steady-state activation profile is hyperpolarized, and as such enables Ca^2+^ entry when L-type Ca^2+^ channels are deactivated. In theory, Ca^2+^ entry via T-type Ca^2+^ channels could impact tone development by directly contributing to the cytosolic Ca^2+^ pool or by acting locally and indirectly to trigger Ca^2+^ waves. Ca^2+^ waves are slow asynchronous events that spread from end to end and whose triggering is tied to the opening of sarcoplasmic reticulum IP_3_Rs by IP_3_ and Ca^2+^^24^. Using a Ca_V_3.1^−/−^ model, we tested whether Ca^2+^ entry through this particular T-type channel does indeed facilitate myogenic tone at hyperpolarized voltages and if this functional response is coupled to the governance of Ca^2+^ waves.

Our examination of Ca_V_3.1 began with experiments to confirm the absence of Ca_V_3.1 in mesenteric arterial smooth muscle cells from genetic deletion mice (Fig. 1). Three approaches were used, the first being PCR which confirmed Cacna1g gene (Ca_V_3.1) modification in Ca_V_3.1^−/−^ mice. Second, protein analysis using immunohistochemistry showed that surface expression of Ca_V_3.1 was notably lacking in Ca_V_3.1^−/−^ but not C57BL/6 cells. These observations aligned with the results from the third, functional approach (whole-cell electrophysiology), which revealed that the nifedipine/Ni^2+^ resistant Ba^2+^ current, previously ascribed to Ca_V_3.1^21,22^ was also absent in mesenteric arterial smooth muscle cells harvested from genetic deletion mice. The absence of this T-type Ca^2+^ channel coincided with a reduction in systolic and diastolic blood pressure, a finding consistent with a role in hemodynamic control. Past observations are limited, with one study reporting no difference in blood pressure, although values were unrealistically low for both Ca_V_3.1^−/−^ and C57BL/6 mice^26^. A second showed blood pressure trending lower in Ca_V_3.1^−/−^ mice, along with a more substantive reduction in blood pressure variability^27^. While the mechanism driving the blood pressure change is unclear, it’s reasonable to assert a role for diminished myogenic tone, an idea we tested in isolated mesenteric arteries across a full pressure range. Consistent with expectations, a reduction in myogenic tone was observed in Ca_V_3.1^−/−^ arteries, particularly at lower pressure (Fig. 2) when vessels are hyperpolarized and T-type Ca^2+^ channels more active in the steady state^28^. In considering these observations, prudent controls are key, the first being an assessment of an artery’s passive structural properties. In this regard, we observed no change in the arterial distensibility in vessels harvested from Ca_V_3.1^−/−^ or C57BL/6 mice. Likewise, in a second set of controls, this study did not observe a change in arterial contractility to PE across a full concentration range in the absence or presence of nifedipine, an L-type Ca^2+^ channel blocker (Fig. 3). These results confirm that the molecular machinery mediating PE-induced constriction remains intact in Ca_V_3.1^−/−^ animals, as does the signalling pathways downstream from the α_1_-adrenoreceptor. Past studies have performed similar agonist controls and findings have been somewhat conflicted, with Ca_V_3.1 deletion notably reducing mesenteric arterial responsiveness in one study^29^, yet having the markedly opposite effect in another, presumptively increasing the Ca^2+^ sensitivity of the contractile apparatus^20^.

In contextualizing the preceding observations, one should recognize past inferential work linking T-type Ca^2+^ channels to myogenic tone using pharmacology with known off-target effects. This approach typically entailed the probing of myogenic tone at rest and in the presence of an L-type Ca^2+^ channel blocker to isolate residual tone whose sensitivity to T-type Ca^2+^ channel inhibition was then tested^12,30,31^. One should also consider past work with Ca_V_3.1^−/−^ mice^16^ highlighting a role for Ca_V_3.1 channels in tone development (at low pressure), although without defining mechanism^20^. Finally, in using this genetic deletion model, acknowledgement of other cardiovascular effects is key, in particular bradycardia^26^ and impaired blood pressure regulation through impaired NO formation^32^.

Ca^2+^ waves are slow-spreading, end-to-end events initiated by a stimulus that drives the release of Ca^2+^ from the sarcoplasmic reticulum^33,34^. The initiation and spread of these asynchronous events are tied to IP_3_R, Ca^2+^-permeable channels whose activation depends on IP_3_ and Ca^2+^ binding to cytosolic sites^25^. Past work in rat cerebral arteries has shown that Ca^2+^ waves are present at low intravascular pressure and that frequency rises as pressure is elevated into the lower physiological range^24^. Pharmacological attenuation of Ca^2+^ waves through IP_3_R blockade or impairment of store refilling results in diminished pressure-induced constriction particularly at low intravascular pressure when arteries are more hyperpolarized^24^. Respectful of these results, it follows that low threshold Ca_V_3.1 channels provide the Ca^2+^ needed to trigger Ca^2+^ waves and foster myogenic tone when L-type Ca^2+^ channels are decidedly less active. This concept was tested three ways, the first examining Ca^2+^ wave generation in Ca_V_3.1^−/−^ arteries, the second ascertaining if Ca_V_3.1 colocalized with IP_3_Rs, and the final determining if Ca^2+^ wave inhibition in C57BL/6 mice results in a Ca_V_3.1^−/−^ contractile phenotype. Findings in Fig. 4 first reveal that Ca^2+^ wave generation is robust in control mesenteric arteries as defined by the number of firing cells and the rate of Ca^2+^ waves per firing cell. Analogous to past work in rat cerebral arteries, nifedipine didn’t impact Ca^2+^ wave generation, consistent with L-type Ca^2+^ channels playing little role in initiating or maintaining these events^24^. Ca^2+^ waves were significantly reduced in Ca_V_3.1^−/−^ arteries and abolished in control arteries by 2-APB, and xestospongin C, IP_3_R inhibitors, findings consistent with this T-type Ca^2+^ channel driving sarcoplasmic reticulum dependent events. These intriguing findings aligned with results from the PLA that note a close spatial association between Ca_V_3.1 and IP_3_R. In detail, this assay involves the binding of primary antibodies to two target proteins and then uses secondary antibodies with conjugated DNA strands which form a circular DNA template for amplification if proteins are < 40 nm apart^10^. The amplified product, detected as bright red puncta, is clearly visible in Fig. 5, thus, it is logical to conclude that Ca^2+^ flux via Ca_V_3.1 should be sufficient to open IP_3_Rs. In light of both results, final experiments assessed whether reduced Ca^2+^ wave production in C57BL/6 vessels generate a functional phenotype akin to Ca_V_3.1^−/−^ arteries. In this regard, we monitored myogenic tone in mesenteric arteries (as a percentage; C57BL/6 and Ca_V_3.1^−/−^) at rest and following treatment with nifedipine alone or with 2-APB or xestospongin C (Fig. 6). We observed residual tone in nifedipine-treated C57BL/6 arteries but not Ca_V_3.1^−/−^ arteries, a difference that could be abolished, particularly at low intravascular pressures (20–40 mmHg) through IP_3_R blockade. This loss of tone parallels a similar loss in tone, and likewise Ca^2+^ waves in C57BL/6 mice when kurtoxin, a Ca_V_3.x blocker was applied on top of nifedipine, an L-type Ca^2+^ channel blocker (Fig. 7). While some caution is warranted when drawing a relationship between Ca^2+^ waves and myogenic tone, the preceding interpretation does align with other published observations. They include: (1) 2-APB treatment having no impact on global [Ca^2+^] while reducing myogenic tone^35^; (2) 2-APB only dilating arteries which prior to treatment were generating Ca^2+^ waves^36^; and (3) Ca^2+^ waves abrogation corelating with reduced myosin light chain phosphorylation particularly at lower pressure^24^.

Two final points in this study require further consideration. First, while differences in myogenic tone between Ca_V_3.1^−/−^ and C57BL/6 arteries were evident at lower pressures (20–60 mmHg), the same trend was present at higher pressures, although without statistical significance. This finding is perhaps unsurprising as L-type Ca^2+^ channels rise to dominate [Ca^2+^]_i_ as arteries depolarize with pressurization. Second, while our work noted Ca^2+^ wave insensitivity to L-type Ca^2+^ channel blockade, like the cerebral vasculature^24^, it lies in contrast to cremaster arterioles where nifedipine attenuated Ca^2+^ wave formation^37^. This discrepancy suggests there may be mechanistic uniqueness among vascular beds, which to date is unappreciated. Alternatively, one could potentially argue the higher concentration of nifedipine (1 μM) used on cremaster arteries may be blocking Ca_V_3.1 and consequently the triggering of IP_3_R^38–40^. This perspective is consistent with electrophysiology observations noting that T-type Ca^2+^ channels are partially blocked by low micromolar nifedipine^41,42^.

## Conclusion

**Figure 8:**
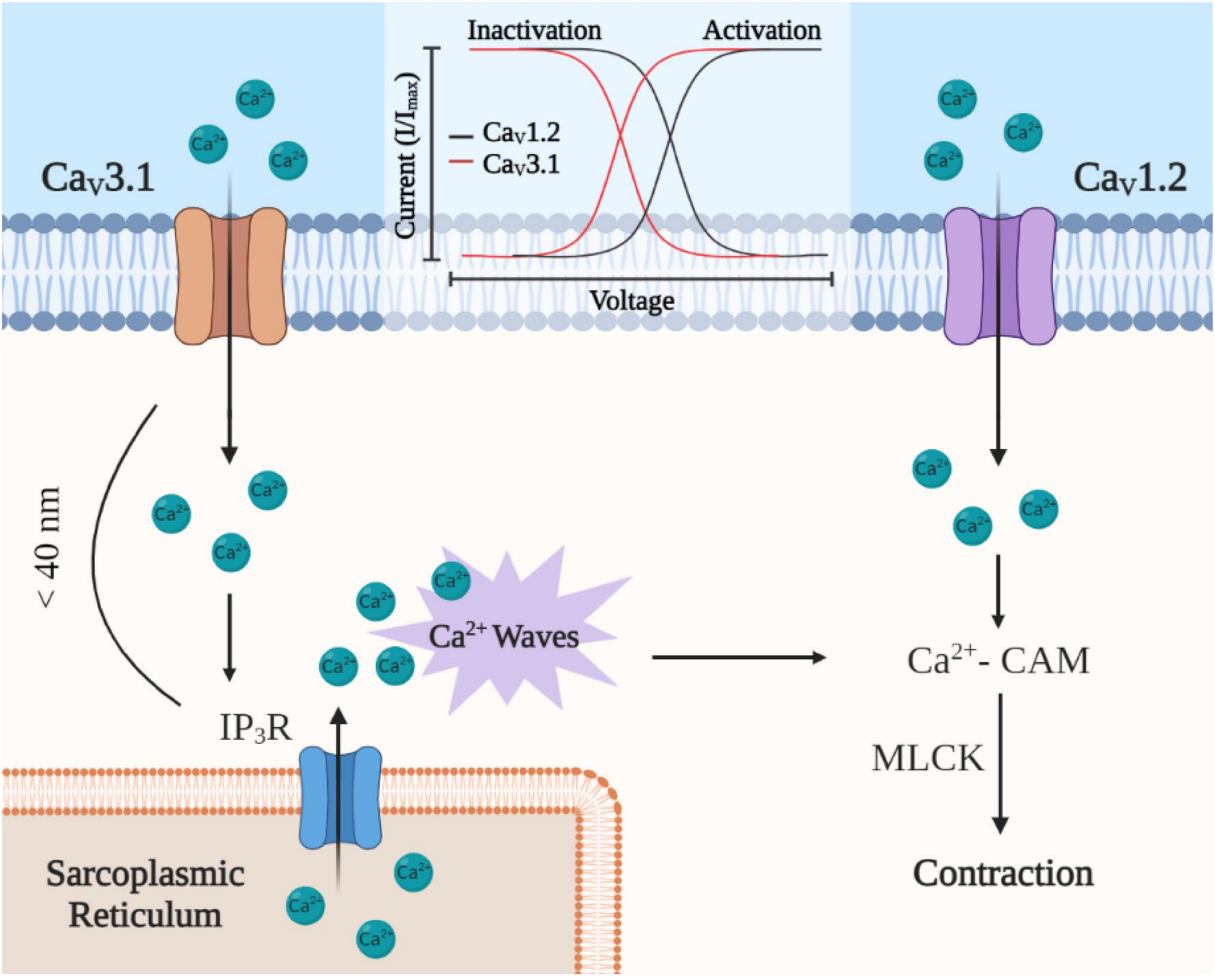
High-voltage-activated Ca_V_1.2 channels control [Ca^2+^]_i_ when intravascular pressure is elevated and membrane potential depolarized. In contrast, Ca_V_3.1 channels display a hyperpolarized profile with more negative activation/inactivation properties compared to Cav1.2 channels. Ca_V_3.1 channels foster Ca^2+^ wave generation likely through sarcoplasmic reticulum IP_3_R activation as the two proteins lie in close proximity. Ca^2+^ waves are known to induce a Ca^2+^-calmodulin (CAM)-dependent activation of myosin light chain kinase (MLCK) which regulates myosin phosphorylation leading to myogenic tone control. Ca_V_3.1 deletion is coupled to reduced blood pressure and hemodynamic control thus bearing clinical importance. Created with BioRender.com.

This study presents three key findings, summarized in Fig. 8: First, Ca_V_3.1^−/−^ mice have lower blood pressure, and mesenteric arteries display diminished myogenic tone compared to controls. Second, immunohistochemical analysis reveals that Ca_V_3.1 lies within 40 nm of IP_3_R1, and when this arrangement is genetically disrupted, arteries generate fewer Ca^2+^ waves. Third, a pharmacological blockade of IP_3_Rs in C57BL/6 arteries produces a phenotype similar to Ca_V_3.1^−/−^ vessels, that being diminished myogenic tone at lower intravascular pressure. By establishing a clear sequential relationship between Ca_V_3.1, Ca^2+^ waves and myogenic tone, this study advances the understanding of vascular contractility and highlights a new target for therapeutic control. In this regard, one could provocatively suggest that development of selective Ca_V_3.1 blockers could be of value in the management of hypertension.

### Supplementary Information

Supplementary Information.

## Data Availability

All data generated or analysed during this study are included in this published article.

## Abbreviations

DAPI: 4′,6-Diamidino-2-phenylindole
IP_3_: Inositol 1,4,5-trisphosphate
PE: Phenylephrine
PLA: Proximity ligation assay
2-APB: 2-Aminoethoxydiphenyl borate
