# Supplementary material for: CaV3.1 channels facilitate calcium wave generation and myogenic tone development in mouse mesenteric arteries
Source: Sci Rep. 2023 Nov 21;13:20407. doi: 10.1038/s41598-023-47715-3 (PMC10663617; doi:10.1038/s41598-023-47715-3)
Supplement: Supplementary file 1 — Supplementary Information. [file 41598_2023_47715_MOESM1_ESM.docx]

**Supplementary Material**

**I. Supplementary Figure**

**
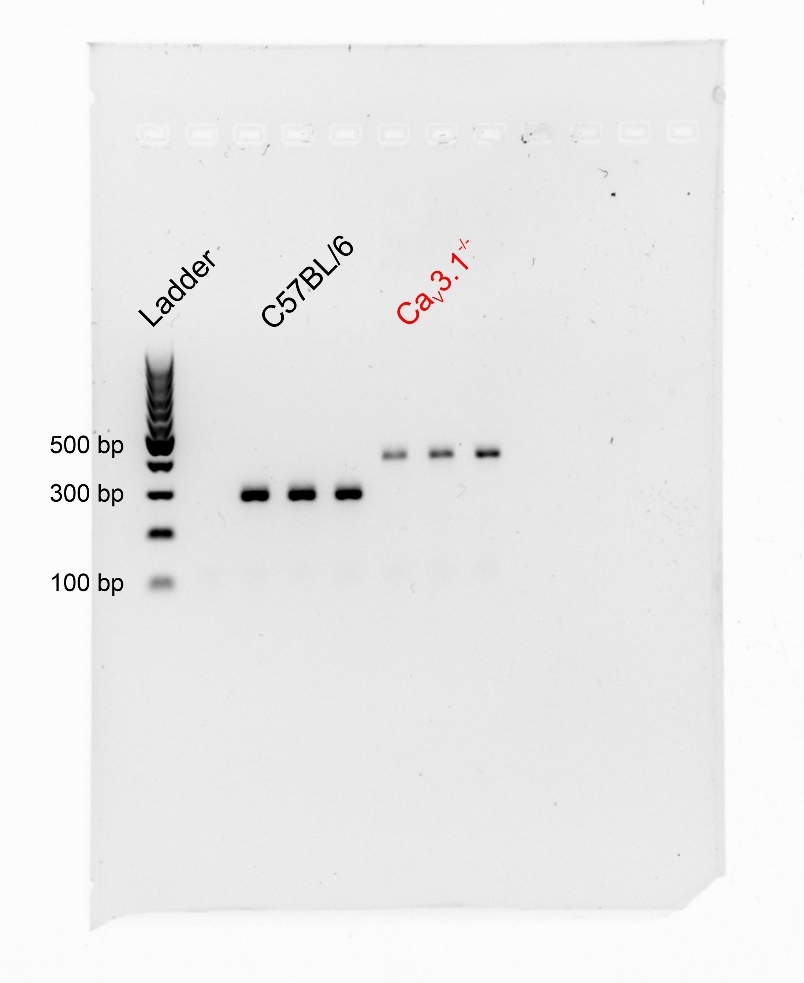
**

**Figure S1. Confirmation of successful modification of** **Cacna1g gene (Ca_V_3.1).** Original uncropped gel from Figure 1Aa is shown. DNA was extracted from ear notches (C57BL/6 and Ca_V_3.1^-/-^ mice) and amplified; the different product sizes confirm the gene modification leading to functional knockout.

**II. Major Resources Table**

**Animals**

| **Species** | **Vendor or Source** | **Strain** | **Background Strain** | **Sex** | **Persistent ID / URL** |
| --- | --- | --- | --- | --- | --- |
| Mouse | Jackson Laboratories | C57BL/6 |  | Males | WT (Jax Strain # 000664) |
| Mouse | In-house breeding colony | C57Bl/6-CaCna1g^tm1Hssh | C57BL/6 | Males | - |

**Antibodies**

| **Target antigen** | **Vendor or Source** | **Catalog #** | **Working concentration** | **Persistent ID / URL** |
| --- | --- | --- | --- | --- |
| Ca_V_3.1 | Alomone Laboratories | ACC-021 | 1:200 | [CACNA1G Antibody (S178A-9) (NBP2-59322): Novus Biologicals](https://www.novusbio.com/products/cacna1g-antibody-s178a-9_nbp2-59322) |
| IP_3_R1 | Alomone Laboratories | ACC-019 | 1:200 | [Anti-IP3 Receptor-1 / IP3R1 / ITPR1 Antibody \| #ACC-019 \| Alomone Labs](https://www.alomone.com/p/anti-ip3-receptor-1/ACC-019) |
| Alexa Fluor 488 Donkey Anti-Rabbit IgG | ThermoFisher | A-21206 | 1:1000 | [Donkey anti-Rabbit IgG (H+L) Highly Cross-Adsorbed, Alexa Fluor™ 488 (A-21206) (thermofisher.com)](https://www.thermofisher.com/antibody/product/Donkey-anti-Rabbit-IgG-H-L-Highly-Cross-Adsorbed-Secondary-Antibody-Polyclonal/A-21206) |
| Rabbit (Duolink® In Situ PLA® Probe Anti-Rabbit PLUS) | Sigma-Aldrich | DUO92002-100RXN | 1:200 | [Duolink In Situ PLA Probe Anti-Rabbit PLUS Sigma (sigmaaldrich.com)](https://www.sigmaaldrich.com/CA/en/product/sigma/duo92002) |
| Mouse (Duolink® In Situ PLA® Probe Anti-Mouse MINUS) | Sigma-Aldrich | DUO92004-100RXN | 1:200 | [Duolink In Situ PLA Probe Anti-Mouse MINUS Sigma (sigmaaldrich.com)](https://www.sigmaaldrich.com/CA/en/product/sigma/duo92004) |

**Other**

| **Description** | **Source / Repository** | **Persistent ID / URL** |
| --- | --- | --- |
| Fluo-8 | Abcam – ab142773 | [Fluo-8 AM, green fluorescent calcium binding dye (CAS 1345980-40-6) (ab142773) (abcam.com)](https://www.abcam.com/fluo-8-am-green-fluorescent-calcium-binding-dye-ab142773.html?gclsrc=aw.ds\|aw.ds&gclid=Cj0KCQiA54KfBhCKARIsAJzSrdphPgBC69NBxt1S03sYH3ownoWlcmNH8eTDUU4ygcqsVmEWl77gtcMaApg4EALw_wcB) |
| 2-APB | Thermofisher – 524-95-8 | [2-APB, Tocris Bioscience, Quantity: 10mg \| Fisher Scientific](https://www.fishersci.com/shop/products/2-apb-tocris-2/122410) |
| Nifedipine | Sigma-Aldrich – 21829-25-4 | [Nifedipine =98 HPLC,powder 21829-25-4 (sigmaaldrich.com)](https://www.sigmaaldrich.com/CA/en/product/sigma/n7634) |
| PE HCl | Sigma-Aldrich | [Phenylephrine hydrochloride - (R)-(−)-Phenylephrine hydrochloride, (R)-(−)-1-(3-Hydroxyphenyl)-2-methylaminoethanol hydrochloride (sigmaaldrich.com)](https://www.sigmaaldrich.com/CA/en/substance/phenylephrinehydrochloride2036761767) |
| Duolink® In Situ Mounting Medium with DAPI | Sigma-Aldrich – DUO82040 | [Duolink In Situ Mounting Medium with DAPI Sigma (sigmaaldrich.com)](https://www.sigmaaldrich.com/CA/en/product/sigma/duo82040) |
| Duolink® In Situ Detection Reagents Red | Sigma-Aldrich – DUO92008 | [Duolink In Situ Detection Reagents Red Sigma (sigmaaldrich.com)](https://www.sigmaaldrich.com/CA/en/product/sigma/duo92008) |
| Duolink® In Situ Wash Buffers, Fluorescence | Sigma-Aldrich – DUO82049 | [Duolink In Situ Wash Buffers, Fluorescence Sigma (sigmaaldrich.com)](https://www.sigmaaldrich.com/CA/en/product/sigma/duo82049) |
| Donkey serum | Sigma-Aldrich – D9663 | [Donkey serum - 10 ML size (sigmaaldrich.com)](https://www.sigmaaldrich.com/CA/en/product/sigma/d9663?gclid=Cj0KCQiA54KfBhCKARIsAJzSrdqLUDdMBHtcs66xsScnx7Dp68IhH8tpoPmo18cQcaACtiyw9Weyy3UaArROEALw_wcB&gclsrc=aw.ds) |
| Tween 20 | Sigma-Aldrich – 85113 | [Tween™ 20 Surfact-Amps™ Detergent Solution (thermofisher.com)](https://www.thermofisher.com/order/catalog/product/85113?ef_id=Cj0KCQiA54KfBhCKARIsAJzSrdrb_EakH5xRqlhvuZKUjQrA_xuSHISCK_F1YGGauL8mDpw25ol_3ZIaAjD6EALw_wcB:G:s&s_kwcid=AL!3652!3!606658602131!e!!g!!tween%2020%20sigma!13368767892!123500397976&cid=bid_pca_ppf_r01_co_cp1359_pjt0000_bid00000_0se_gaw_con_con&gclid=Cj0KCQiA54KfBhCKARIsAJzSrdrb_EakH5xRqlhvuZKUjQrA_xuSHISCK_F1YGGauL8mDpw25ol_3ZIaAjD6EALw_wcB#/85113) |
| Paraformaldehyde | Sigma-Aldrich – 30525-89-4 | [Paraformaldehyde \| Sigma-Aldrich (sigmaaldrich.com)](https://www.sigmaaldrich.com/CA/en/search/paraformaldehyde?focus=products&page=1&perpage=30&sort=relevance&term=paraformaldehyde&type=product_name) |
| Mountant with DAPI | Thermofisher – P36971 | [ProLong™ Diamond Antifade Mountant with DAPI (thermofisher.com)](https://www.thermofisher.com/order/catalog/product/P36971) |
| Kurtoxin | Sigma-Aldrich – K1514 | [Kurtoxin \| Sigma-Aldrich (sigmaaldrich.com)](https://www.sigmaaldrich.com/CA/en/product/sigma/k1514) |
| QIAamp Fast DNA Tissue Kit | Qiagen – 51404 | [QIAamp Fast DNA Tissue Kit (qiagen.com)](https://www.qiagen.com/us/products/discovery-and-translational-research/dna-rna-purification/dna-purification/genomic-dna/qiaamp-fast-dna-tissue-kit) |
